# Research on the Genetic Polymorphism and Function of *inlA* with Premature Stop Codons in *Listeria monocytogenes*

**DOI:** 10.3390/foods14172955

**Published:** 2025-08-25

**Authors:** Xin Liu, Binru Gao, Zhuosi Li, Yingying Liang, Tianqi Shi, Qingli Dong, Min Chen, Huanyu Wu, Hongzhi Zhang

**Affiliations:** 1 School of Health Science and Engineering, University of Shanghai for Science and Technology, Shanghai 200093, China; 2 Shanghai Municipal Center for Disease Control and Prevention, Shanghai 200336, China

**Keywords:** *inlA*, clonal complex types, *Listeria monocytogenes*, virulence, PMSC mutation

## Abstract

*Listeria monocytogenes* is a Gram-positive bacterial species that causes listeriosis, a major foodborne disease worldwide. The virulence factor *inlA* facilitates the invasion of *L. monocytogenes* into intestinal epithelial cells expressing E-cadherin receptors. Naturally occurring premature stop codon (PMSC) mutations in *inlA* have been shown to result in the production of truncated proteins associated with attenuated virulence. Moreover, different *L. monocytogenes* strains contain distinct *inlA* variants. In this study, we first characterized *inlA* in 546 *L. monocytogenes* strains isolated from various foods in Shanghai. The results showed that 36.1% (95% Confidence Interval: 32.0~40.2%) of the food isolates harbored *inlA* with PMSC, which was found to be associated with clonal complex (CC) types, with the highest proportions observed in CC9 and CC121. To investigate the function of *inlA*, we first used the dominant CC87 isolated from patients as the test strain and constructed an *inlA-deleted* strain via homologous recombination. Resistance tests and virulence tests showed that while *inlA* did not affect the resistance of *L. monocytogenes*, it significantly influenced cell adhesion and invasiveness. To further explore the function of *inlA*, we performed virulence tests on five CC-type strains carrying *inlA* with PMSC and their corresponding strains with intact *inlA*. We found that the virulence of *L. monocytogenes* strains carrying *inlA* or *inlA* with PMSC was associated with their CC type. Our preliminary results showed that premature termination of *inlA* did not significantly affect the adhesion and invasion abilities of low-virulence CC-type *L. monocytogenes* strains in Caco-2 cells, but substantially promoted those of high-virulence strains such as CC8 and CC7. In summary, this study preliminarily evaluated the effects of *inlA* integrity and PMSC mutation variation on the virulence of *L. monocytogenes*, providing a foundation for further research on *inlA*-related pathogenic mechanisms.

## 1. Introduction

*Listeria monocytogenes* causes zoonotic listeriosis in a broad range of hosts, including humans and various animals. *L. monocytogenes* primarily enters the intestinal tract through contaminated food, subsequently triggering host infections. Infection mainly causes gastroenteritis, meningitis, encephalitis, and miscarriage, with a mortality rate of 20–30% [1,2]. About 1600 people contract listeriosis annually through contaminated food [3]. In 2023, the EU reported 2952 confirmed cases and 335 deaths due to *L. monocytogenes* [4]. In China, 147 sporadic and 82 outbreak cases were recorded between 1964 and 2010, with an overall mortality rate of 26% (46% in neonatal cases) [5]. In addition, a 2011–2017 review found 562 cases (higher than the previous decade), with 23.78% mortality in non-perinatal patients and 32.68% of perinatal patients experiencing miscarriage/neonatal death [6]. China’s Listeria burden may be underestimated due to inadequate monitoring [7].

Host infections by *L. monocytogenes* involve the collaborative action of multiple genes encoding virulence factors, predominantly the internalin protein family. These factors mediate the internalization of *L. monocytogenes* into host nonphagocytic cells and cross three major barriers in the host: the intestinal, blood-brain, and maternal-fetal placental barriers [8]. InlA is the earliest identified and most thoroughly studied internalin protein, which mediates the invasion of host cells by *L. monocytogenes* and remains one of the most thoroughly studied virulence factors to date [9]. Research has shown that the expression of full-length *inlA* is one of the primary virulence factors enabling *L. monocytogenes* to cross the intestinal barrier and invade epithelial cells [10]. The N-terminal domain of this protein contains leucine-rich repeats (LRRs). Through specific binding to the host cell receptor E-cadherin, InlA induces local polymerization of the host actin cytoskeleton, thereby promoting bacterial internalization [10].

However, point mutations in the *inlA* gene may lead to premature stop codons (PMSCs), generating truncated InlA, a secretory protein that cannot be anchored to the bacterial cell wall [11]. Studies have shown that this truncation of *inlA* reduces the ability of *L. monocytogenes* to invade human intestinal epithelial cells in vitro [12]. For example, one study demonstrated that guinea pigs challenged with *inlA*-PMSC strains had a median infectious dose approximately 1.2–1.3 log higher than those challenged with epidemic clonal strains [13]. However, certain strains containing *inlA* with PMSC can also be pathogenic [14,15]. Analysis of the *inlA* sequence of *L. monocytogenes* isolates revealed that strains containing PMSCs at position 976 were more invasive than those without PMSCs [16]. These results indicate that there is no direct correlation between the *inlA* sequence or InlA integrity and the ability to invade cells or cause infection. For this reason, it is crucial to verify not only the presence or absence of *inlA* mutations, but also the types of PMSC mutations to identify potential pathogenic risks.

*L. monocytogenes* strains exhibit considerable diversity in virulence-related phenotypes, including highly and low-virulent clones. Most clinical isolates have intact *inlA*, while *inlA* with PMSCs is more prevalent in environmental and food-related isolates [17]. Currently, many types of PMSC mutations in InlA have been documented by Li et al. [11]. Moreover, there is a strong correlation between the major sequence types (STs) and *inlA* alleles. For example, ST1 and ST2 strains are associated with *inlA*_3, whereas ST6 strains are associated with *inlA*_8 [11]. However, early studies on *L. monocytogenes* InlA only focused on the presence of PMSCs [14,18], and existing research on *inlA* with PMSC has analyzed a relatively small number of strains, mainly concentrating on certain specific types or regions of *L. monocytogenes* isolates. The impact of different types of PMSCs in *inlA* on the pathogenicity of *L. monocytogenes* has not been thoroughly studied.

Therefore, this study included 546 *L. monocytogenes* strains isolated from various foods in Shanghai as the research objects. Whole-genome sequencing (WGS) was used to analyze the diversity of truncated *inlA* and its relationship with clonal complex (CC) type. Furthermore, resistance assays and virulence experiments were conducted to investigate the functions of intact *inlA* and truncated *inlA*. A comprehensive study on the pathogenicity of *inlA* in PMSCs can provide a reference for a deeper understanding of the pathogenic mechanisms related to *inlA*.

## 2. Materials and Methods

### 2.1. Strain and Cultivation Conditions

A total of 546 strains isolated from different types of foods, including pork, chicken, processed meat products, beef, lamb, cooked meat products, ready-to-eat (RTE) food, seafood products, and duck, were used in this study. The presence of *L. monocytogenes* in these foods was determined according to the Chinese national standard [19]. The reference strain was *L. monocytogenes* isolate ATCC19115. The strain information used in the virulence and resistance experiments is shown in Table 1. All bacteria were stored in trypticase soy broth with 0.6% yeast extract (TSB-YE) supplemented with 25% glycerol at −80 °C. Each strain was initially isolated on tryptic soy agar plates with 0.6% (*w/v*) yeast (TSA-YE) (Hopebio Technology Co. Ltd., Qingdao, China) and incubated at 37 °C for 24 h. These working stocks were stored at 4 °C and renewed monthly. For experiments, each strain was transferred to TSB-YE and incubated overnight at 37 °C to obtain late stationary phase cells, ca. 10^9^ CFU/mL.

### 2.2. Whole Genome Sequence (WGS)

WGS was performed on all 546 *L. monocytogenes* isolates. Briefly, after overnight culture, genomic DNA was extracted using the DNeasy Blood & Tissue Kit (QIAGEN, Hilden, Germany) according to the manufacturer’s protocol with a minor modification (prelysis using lysozyme for 30 min). DNA concentration, quality, and integrity were assessed using a Qubit Fluorometer (Thermo Scientific, Waltham, MA, USA). Short-read sequencing was performed on an Illumina HiSeq platform (Illumina, San Diego, CA, USA) at an external facility, and the sequence data were evaluated using FastQC v0.11.2 (Cambridge, London, UK). After de novo assembly of the short-read raw sequences of *L. monocytogenes* isolates using CLC Genomics Workbench v7.0 (CLC Bio, Aarhus, Denmark), the sequences were trimmed using Trimmomatic v0.36 and then assembled using BioNumerics v7.6 (Applied Maths, Kortrijk, Belgium) before further analysis. Multilocus Sequence Typing (MLST) and CCs were deduced in silico from the genome sequences using the BIGSdb platform (https://bigsdb.pasteur.fr/cgi-bin/bigsdb/bigsdb.pl?db=pubmlst_listeria_seqdef&page=sequenceQuery, accessed on 5 March 2025). In silico deduction of *inlA* from the genome sequences was achieved using the BIGSdb platform (https://bigsdb.pasteur.fr/cgi-bin/bigsdb/bigsdb.pl?db=pubmlst_listeria_seqdef&page=sequenceQuery, accessed on 5 March 2025).

### 2.3. Phylogenetic Analysis of inlA with PMSC

A phylogenetic tree comprising 197 *inlA* genes was constructed using MEGA (version 11) software, using the *inlA* gene of the reference strain EGD-e as the baseline, and was visualized using the Evolview platform (https://www.evolgenius.info/evolview, accessed on 20 March 2025). The nucleotide sequence of *inlA* of EGD-e was obtained from the NCBI database (https://www.ncbi.nlm.nih.gov/, accessed on 20 March 2025).

### 2.4. Construction of inlA Deletion Strain

To verify the effect of *inlA* on the virulence of *L. monocytogenes*, the CC87-type strain LM119 was used as a positive control. Using pLR16-pheS* as the plasmid, an *inlA* gene deletion strain was constructed via homologous recombination. The specific procedures were as follows: Using the genomic DNA of LM119 as a template, the upstream and downstream homologous arm fragments of *inlA* were amplified using the primer pair, up-*inlA*. F/up-*inlA*.R and down-*inlA*.F/down-*inlA*.R. Then, the upstream and downstream homologous arm fragments of *inlA* were used as templates to amplify the upstream and downstream fragments with approximately 20 bp homologous arms using primers 2up-*inlA*. F/2up-*inlA*.R and 2down-*inlA*.F/2down-*inlA*.R, respectively. These fragments were then used as templates for overlap PCR to amplify the fusion fragment of the upstream and downstream homologous arms of the *inlA* gene. Using the fusion fragment AB as a template, the fusion fragment 2AB with approximately 20 bp homologous arms of the vector was amplified using the primers AB-16.F/R. The pLR16-pheS* plasmid was double-digested with the restriction endonucleases KpnI and XhoI. Subsequently, the fusion fragment 2AB was ligated into the digested vector pLR16 pheS* using a seamless cloning kit and then transferred into *Escherichia coli* DH5α competent cells. The transformed cells were spread on LB with ChL (25 µg/mL)plates for culture and screening. After sequencing and alignment, the recombinant plasmid pLR16-pheS*-Δ*inlA* was constructed.

After preparing competent LM119 cells as previously described [20], the recombinant plasmid was transformed into these cells (2.5 kV, 2.5 μF, 200 Ω) by electroporation. Positive clones were screened, and the Δ*inlA* deletion strain was confirmed by PCR identification. The upstream and downstream sequences and target genes were detected using primers up-*inlA*. F/down-*inlA*.R and *inlA*.F/R, respectively, with the wild-type LM119 strain as a control. The deletion strain was inoculated into BHI medium and continuously cultured for approximately ten generations. PCR identification of the wild-type and deletion strains was performed using the primer pair, up-*inlA*. F/down-*inlA*.R and *inlA*.F/R primers were used to confirm the genetic stability of the Δ*inlA* deletion strain. Finally, the obtained Δ*inlA* deletion strain, named LM119-Δ*inlA*, was stored for subsequent experiments. The primers used in this study are listed in Supplementary Table S1.

### 2.5. Growth Experiment

Stationary-phase bacterial cultures of LM119 and LM119-Δ*inlA* were adjusted to 10^3^ CFU/mL using TSB-YE. Then, 200 μL of the bacterial solutions from different treatments were added to a 100-well honeycomb plate, with 200 μL of TSB-YE used as the blank control. The plate was placed in a Bioscreen C incubator (Oy Growth Curves Ab Ltd., Helsinki, Finland) and cultured at 30 °C. The OD_600_ value was measured every 30 min. The plate was shaken for 20 s before each measurement to ensure thorough mixing.

### 2.6. Biofilm Formation Assay

The experiment was performed as described previously [21]. Using LM119 as a control, the biofilm-forming ability of LM119-Δ*inlA* on stainless steel sheets was tested. Stainless steel (SS; 14 mm × 12 mm × 1 mm; Type 304) was submerged in absolute ethanol overnight to remove grease and oils. After rinsing with distilled water, the stainless steelsheets were dried and autoclaved at 121 °C for 15 min. In 24-well plates, each well was filled with 2 mL of bacterial suspension (10^4^ CFU/mL) to submerge one SS per well. The plates were incubated at 25 °C for 24, 48, 72, 96, and 120 h and at 37 °C for 24 and 48 h. Negative controls consisted of SS incubated in sterile medium. After incubation, the sheets were rinsed thrice with 0.85% NaCl solution to remove unattached cells and then transferred to centrifuge tubes containing 3 mL of normal saline and a suitable amount of sterilized glass beads. To detach the biofilm cells from the surface, the tubes were vortexed using a vortex shaker and agitated manually for 2 min. The resulting suspensions were serially diluted 10-fold, plated on TSA-YE, and incubated at 37 °C for 24 h. The results were expressed as Log CFU/cm^2^. Each experiment was repeated three times, starting from independent bacterial cultures.

### 2.7. Simulated Gastrointestinal Tract Experiment

The simulated gastrointestinal (GI) system consisted of a continuous process comprising simulated oral saliva (SOS), simulated gastric fluid (SGF), and simulated intestinal fluid (SIF). The specific experimental procedures were adapted from the method described by Cheng et al. [22], with some modifications. First, SOS, SGF, and SIF were preheated and maintained at 37 °C in a water bath before the experiment. For each strain, 1 mL of the 24-h culture was centrifuged at 12,000× *g* for 2 min to discard the supernatant, and the bacterial cells were suspended in 1 mL of 0.85% sodium chloride. The initial bacterial count (X_0_) was determined and served as the initial concentration for the treatment mixed with SOS. Additionally, another 1 mL of the 24-h culture was centrifuged, resuspended in 1 mL of SOS, maintained at 37 °C for 1 min, and then counted. The result (X_1_) represents the number of surviving *L. monocytogenes* cells after treatment with SOS and serves as the initial concentration for subsequent mixing with SGF. Next, 0.5 mL of the sample after SOS treatment was immediately added to 0.5 mL of SGF, incubated at 37 °C for 1 h, and then counted (X_2_). This value represented the number of surviving cells after SGF treatment and served as the initial concentration for mixing with SIF. After the SGF stage, 0.5 mL of the sample was rapidly added to 0.5 mL of SIF, incubated at 37 °C for 2 h, and counted (X_3_), representing the number of surviving cells after SIF treatment. The tolerance of each *L. monocytogenes* strain to SOS, SGF, and SIF was expressed as the logarithmic reduction in microbial counts at each corresponding stage. Each treatment for each strain was set with three replicates and parallels.

### 2.8. In Vitro Virulence Assay

A virulence assay was performed to evaluate the adhesiveness and invasiveness of *L. monocytogenes* to Caco-2 cells, as described in a previous study [23]. Briefly, Caco-2 cells were first resuscitated and passaged in DMEM containing 10% fetal bovine serum, 1.25% glutamine, and 1.25% penicillin-streptomycin solution. Twenty-four hours before the experiment, the cultured Caco-2 cells were seeded into 12-well tissue culture plates (Greiner Bio-One, Frickenhausen, Germany) and incubated until they reached 90% confluence. Then, 1 mL of 24-h culture was centrifuged (12,000 rpm for 2 min), and the pellet was resuspended in 1 mL of DMEM. After aspirating the complete medium, the wells were washed twice with PBS. Then, 1 mL of DMEM and 10 μL of the prepared bacterial suspension were added to each well, followed by gentle shaking to mix. The mixed bacterial suspension was serially diluted and counted, and the results were recorded as N_0_. The treated 12-well plates were incubated in a cell culture incubator (37 °C, 5% CO_2_) for 1 h. After incubation, the supernatant was discarded, and the wells were washed twice with PBS to remove non-adherent cells before subsequent experiments.

For the adhesion assay, 1 mL of 1% Triton-X reagent was added to each well and allowed to stand for 4 min, followed by repeated pipetting to lyse the cells. The cell suspension from each well was serially diluted and counted, and the results were recorded as N_1_.

For the invasion assay, 1 mL of DMEM containing 100 μg/mL penicillin-streptomycin was added to each well and incubated at 37 °C and 5% CO_2_ for 1 h to kill extracellular bacteria. The supernatant was discarded, and the cell layer was gently washed twice with PBS. Then, 1 mL of 1% Triton X-100 reagent was added to each well, allowed to stand for 4 min, and the cells were lysed by pipetting. The suspension from each well was serially diluted, and the cell count was recorded as N_2_. Each treatment for each strain was set with three replicates and parallels.

Adhesion and invasion efficiencies were calculated using the following equations:

$$Adhesion\;efficiency\;=\;N_{1}/N_{0}\;\times \;100\%$$

$$Invasion\;efficiency=N_{2}/N_{0}\times 100\%$$

where *N*_0_ is the number of *L. monocytogenes* in the initial inoculum (Log CFU/mL); *N*_1_ is the number of *L. monocytogenes* adhering to the cell surface (Log CFU/mL); and *N*_2_ is the number of *L. monocytogenes* invading the cell interior (Log CFU/mL).

### 2.9. Data Analysis

All experiments were performed with three biological replicates. Analysis of variance (ANOVA) was conducted using GraphPad 10.1.0 (GraphPad Software Inc., San Diego, CA, USA). Tukey’s post-hoc test (*p* < 0.05) was performed to determine significant differences between group means.

## 3. Results

### 3.1. Distribution of inlA with PMSC

**Figure 1:**
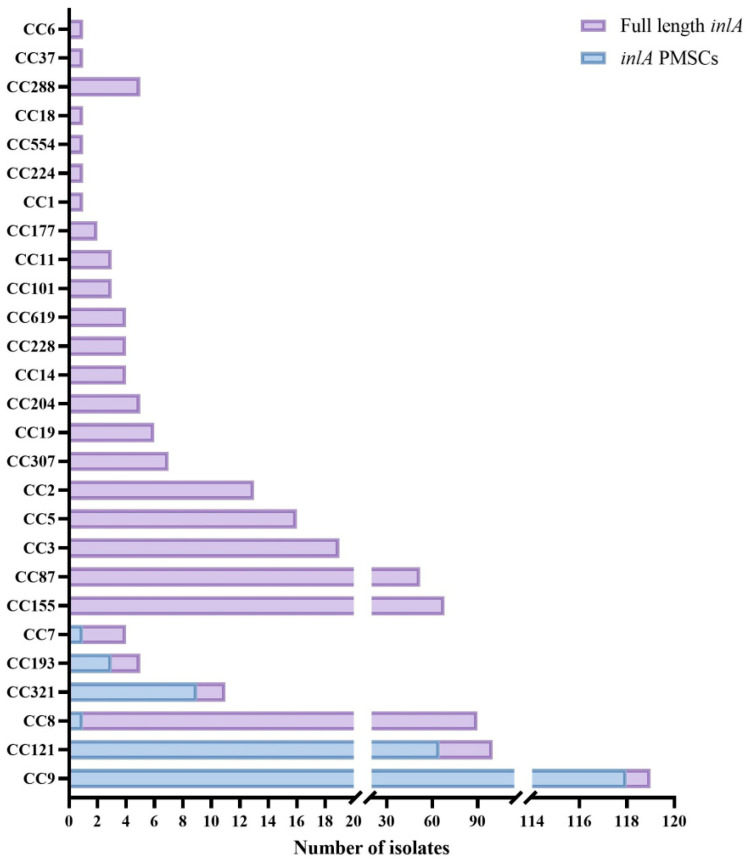
Distribution of *inlA* with premature stop codon (PMSC) in 546 strains of *Listeria monocytogenes* with different CC types.

As shown in Figure 1, this study analyzed 546 food-isolated *L. monocytogenes* strains classified into 27 CCs. All strains carried the *inlA* gene, among which six CCs (22.2%, 6/27) harbored *inlA* with PMSCs, while the remaining 21 CCs harbored *inlA* alleles encoding intact InlA protein. Among the six CCs carrying *inlA* with PMSC, CC9, CC121, CC193, and CC321 exhibited the highest proportions of *inlA* with PMSC, at 99.2% (118/119), 65% (65/100), 60% (3/5), and 81.2% (9/11), respectively. The remaining CCs carrying *inlA* with PMSC were relatively rare, with only one strain each carrying CC8 (1/90) and CC7 (1/4).

### 3.2. Phylogenetic Tree Analysis Based on inlA Gene

**Figure 2:**
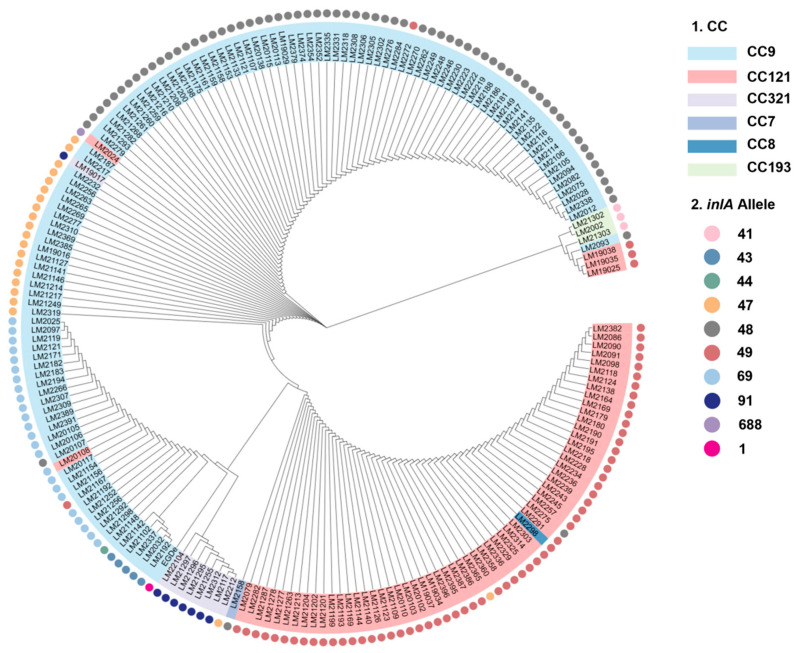
Phylogenetic tree of 197 *L. monocytogenes* isolates based on the nucleotide sequence of *inlA* with PMSC. The right side displays the CCs and *inlA* alleles (represented by circles).

Phylogenetic tree analysis showed that the clustering of *inlA* alleles was largely consistent with the CC type of *L. monocytogenes* (Figure 2). A high degree of consistency was also observed between the *inlA* truncation type and the CC type, indicating that strains with the same *inlA* truncation type have higher homology. For example, in CC9, three types *of inlA*_48, *inlA*_47, and *inlA*_69 were observed. As shown in Figure 2, inlA_48_CC9, inlA_47_CC9, and inlA_69_CC9 clustered with high homology. However, exceptions existed: three strains (3/61) in inlA_49_CC121 had low homology with the other 58 strains. The homology between inlA_91-CC321 and *inlA* was also high.

### 3.3. Relationship Between CCs and Types of inlA with PMSC

The types of *inlA* with PMSC vary among different CC types. As shown in Table 2, the 197 strains carrying *inlA* with PMSC were divided into nine allele types. Among them, CC9 had the most *inlA* with PMSC types, with *inlA*_6, and *inlA*_48 accounting for the highest proportion of 57.6% (68/118), followed by *inlA*_69 (19.5%, 23/118), *inlA*_47 (16.1%, 19/118), *inlA*_43 (4.23%, 5/118), *inlA*_49 (1.69%, 2/118), and *inlA*_44 (0.85%, 1/118). In CC121, *inlA* with PMSC types were divided into five categories, with *inlA*_49 dominating at 93.9% (61/65), followed by *inlA*_48 (1.54%, 1/65), *inlA*_47 (1.54%, 1/65), *inlA*_69 (1.54%, 1/65), and *inlA*_688 (1.54%, 1/65). One CC8 strain with *inlA*-PMSC belonged to *inlA*_49, while a single CC7 strain carried *inlA*_48. CC321 harbored two *inlA*-PMSC types (*inlA*_47 and *inlA*_91), and all CC193 strains were of *inlA*_41. The most abundant types were *inlA*_48 (70 strains), *inlA*_49 (64 strains), *inlA*_69 (24 strains), and *inlA*_47 (21 strains).

Deletion mutations were observed at the 12th base (A) and at position 1637 (A) in the *inlA*_48 and *inlA*_47 allele types, respectively. For *inlA*_49, a C-to-T substitution was observed at position 1474. For *inlA*_69, a G-to-T substitution occurred at position 976, while G-to-A substitutions were observed at positions 1380 and 2054 for *inlA*_43 and *inlA*_44, respectively. For *inlA*_688 and *inlA*_91, C-to-G substitutions were observed at positions 262 and 2100, respectively. Finally, for *inlA*_41, a deletion mutation occurred at position 12 (A). The truncated amino acid lengths after translation ranged from 8 to 691 aa.

### 3.4. Growth of the inlA Deletion Strain

**Figure 3:**
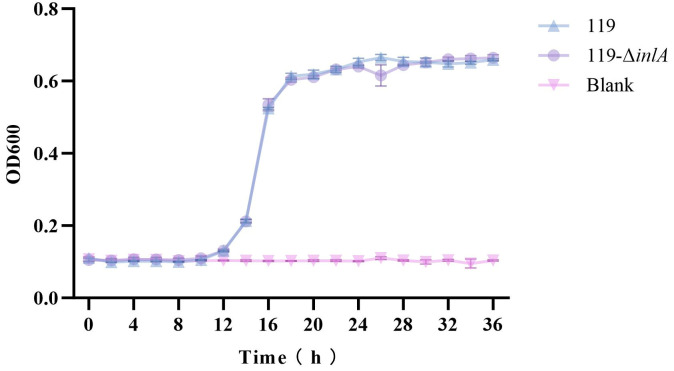
Growth of LM119 d LM119-Δ*inlA* (TSB-YE) at 30 °C.

To evaluate the effect of the *inlA* gene on the growth of *L. monocytogenes*, the growth curves of LM119 and LM119-Δ*inlA* in TSB-YE medium at 30 °C were obtained using sterile TSB-YE medium as a blank control (Figure 3). The results shown in Figure 3 show that LM119 and LM119-Δ*inlA* exhibited similar growth patterns at 30 °C. From 0 to 10 h, the OD_600_ values of both strains remained essentially unchanged. Between 10 and 18 h, the OD_600_ values for both strains increased rapidly from 18 to 26 h. This increase was more gradual from 18 to 26 h, and the values stabilized between 26 and 36 h. At 36 h, the final optical densities of the two strains were 0.66 ± 0.00 and 0.66 ± 0.01, respectively, with no significant differences. These results indicate that the deletion of *inlA* did not affect the growth capacity of LM119.

### 3.5. Biofilm Formation Abilities of the inlA Deletion Strain

**Figure 4:**
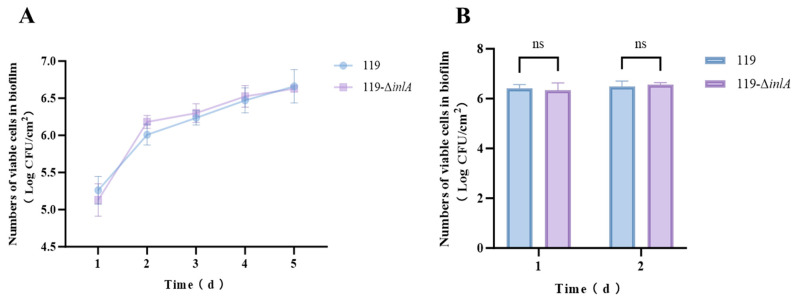
Dynamics of biofilm formation (Log CFU/cm^2^) by LM119 and LM119Δ*inlA* at 25 °C (**A**) and 37 °C (**B**) conditions. Data is presented as the mean ± standard deviation of three independent experiments. “ns” indicates no significant difference.

The biofilm-forming abilities of LM119 and LM119-Δ*inlA* at different temperatures are shown in Figure 4. Both strains exhibited similar biofilm formation trends when cultured at 25 °C for five days (Figure 4A). From day 1 to day 2, biofilm cell growth was the greatest in both strains. The biofilm cell counts of LM119 increased from 5.26 ± 0.19 log CFU/cm^2^ to 6.01 ± 0.14 log CFU/cm^2^, while those of LM119-Δ*inlA* increased from 5.13 ± 0.22 log CFU/cm^2^ to 6.18 ± 0.09 log CFU/cm^2^. From day 2 to day 5, the biofilm cell counts of both strains gradually increased and reached the maximum cell numbers on day 5, at 6.66 ± 0.22 log CFU/cm^2^ and 6.63 ± 0.03 log CFU/cm^2^, respectively. At 37 °C, no significant differences were observed in biofilm formation between LM119 and LM119-Δ*inlA* after 24 and 48 h of culture (Figure 4B). Generally, both strains displayed higher biofilm formation at 37 °C than at 25 °C for the same culture time. However, *inlA* deletion did not affect the biofilm formation ability of *L. monocytogenes* at either temperature.

### 3.6. Results of Continuous GI Treatment on the inlA Deletion Strain

**Figure 5:**
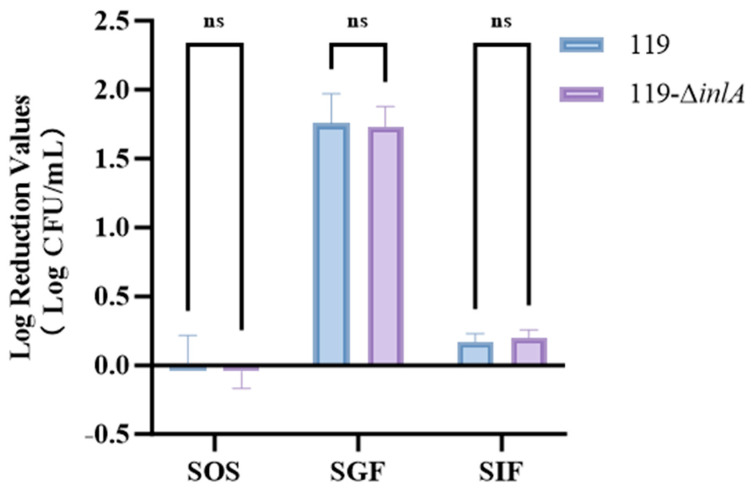
Reduction of populations of LM119 and LM119-Δ*inlA* exposed to continuous SGF. Data is presented as the mean ± standard deviation of three independent experiments. “ns” indicates no significant difference.

The tolerance of *L. monocytogenes* to continuous digestive fluid treatment is shown in Figure 5. The colony count of LM119 remained almost unchanged after treatment in SOS for 1 min, while that of LM119-Δ*inlA* increased slightly (within 0.04 log CFU/mL), but was within the allowable error range and did not indicate bacterial growth in the SOS environment. After treatment in SGF fluid for 1 h, the bacterial concentrations of LM119 and LM119-Δ*inlA* decreased by 1.76 ± 0.21 log CFU/mL and 1.73 ± 0.14 log CFU/mL, respectively, without any significant difference between the strains (*p* ≥ 0.05). After treatment in SIF for 2 h, the bacterial concentrations of LM119 and LM119-Δ*inlA* decreased by 0.17 ± 0.06 log CFU/mL and 0.20 ± 0.06 log CFU/mL, respectively, with no significant differences. Overall, during continuous GI treatment, SGF and SIF exhibited bactericidal capabilities, with SGF demonstrating better bactericidal efficacy. However, SOS exhibited no bactericidal effect on L. monocytogenes. The consistent results observed between the two strains when subjected to continuous GI treatment indicate that the deletion of *inlA* does not affect the strain’s tolerance to GI fluids.

### 3.7. Virulence Assay of the inlA Deletion Strain

**Figure 6:**
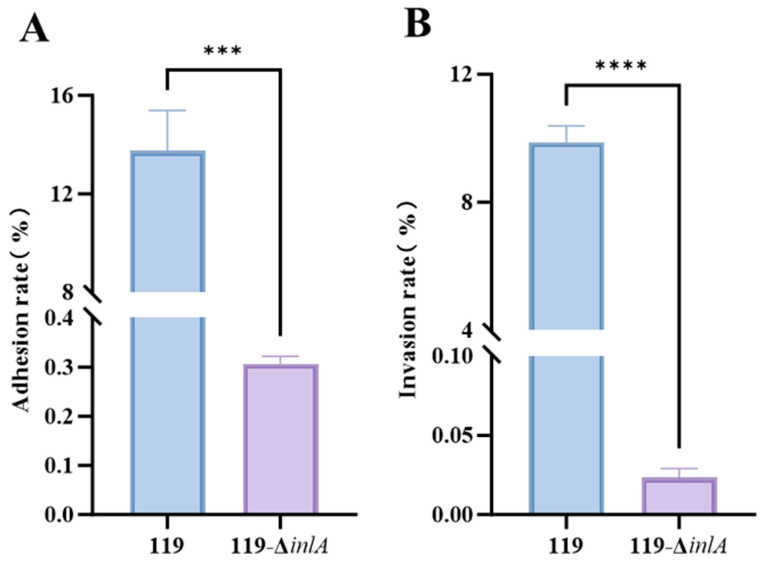
Adhesion (**A**) and invasion (**B**) rates of LM119 and LM119-Δ*inlA* in Caco-2 cells. Data is presented as the mean ± standard deviation of three independent experiments. The *p*-values are as follows: *** *p* < 0.001, **** *p* < 0.0001.

The adhesion and invasion rates of LM119 and LM119—Δ*inlA* to Caco-2 cells are shown in Figure 6. Analysis of the experimental data indicated that the ability of the LM119—Δ*inlA* mutant strain to adhere (Figure 6A) and invade (Figure 6B) Caco-2 cells was significantly lower than that of the LM119 wild-type strain.

### 3.8. Virulence Assay of inlA with PMSC Strains

**Figure 7:**
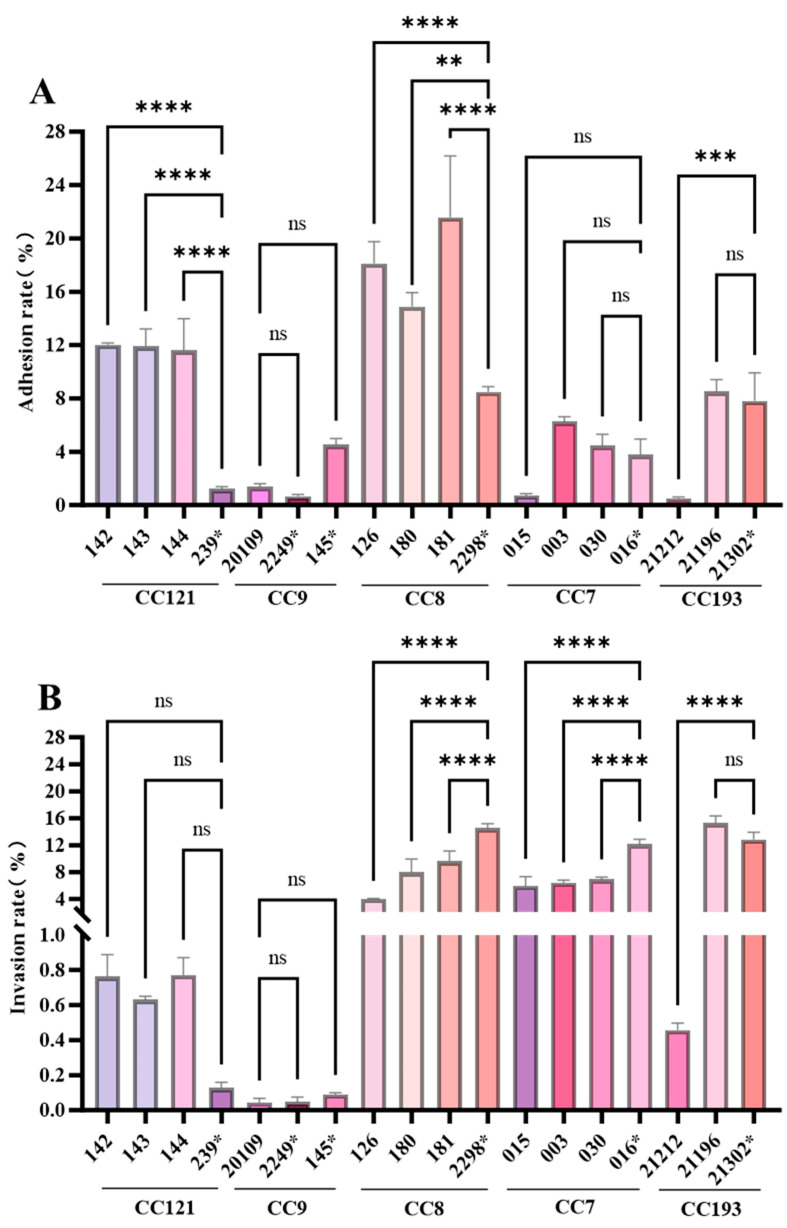
Adhesion (**A**) and invasion (**B**) rates of different CC types of *L. monocytogenes*. Data is presented as the mean ± standard deviation of three independent experiments. “*” Represents the strain carrying *inlA* with PMSC. The *p*-values are as follows: ** *p* < 0.01, *** *p* < 0.001, **** *p* < 0.0001. “ns” indicates no significant difference.

This study investigated how *inlA* (with or without PMSC) in different CC types of *L. monocytogenes* affects Caco-2 cell adhesion and invasion (Figure 7), and our results showed that their virulence is correlated with the CC type.

In CC121 and CC8, all *inlA*-carrying *L. monocytogenes* strains (CC121:142, 143, and 144; CC8:126, 180, and 181) exhibited significantly higher adhesion rates to Caco-2 cells than *inlA*-carrying PMSC-carrying CC121 and CC8 strains (239* and 2298*) (Figure 7A). In CC9 and CC7, there was no significant difference in adhesion rates to Caco-2 cells between strains carrying *inlA* or *inlA* with PMSC (*p* > 0.05). In CC193, the adhesion rate of the *inlA*-carrying strain 21196 to Caco-2 cells showed no significant difference compared to that of *inlA* with the PMSC-carrying strain 21302*. However, the adhesion rate of the *inlA*-carrying strain 21212 to Caco-2 cells was 0.63%, significantly lower than that of the *inlA* with PMSC-carrying strain 21302* (9.64%).

For CC121 and CC9, there was no significant difference in the invasion rates into Caco-2 cells between *inlA*-carrying *L. monocytogenes* and *inlA* PMSC-carrying strains (Figure 7B). In CC8, the invasion rate of the *inlA*-truncated strain 2298* into Caco-2 cells was 14.51%, which was significantly higher than that of the full-length *inlA*-carrying strains 126, 180, and 181 (3.98%, 6.19%, and 9.63%, respectively) (*p* < 0.05). A similar phenomenon was observed in CC7: the invasion rate of the *inlA* with PMSC-carrying strain 016* was 11.94%, while the invasion rates of the *inlA*-carrying strains 015, 003, and 030 were 5.09%, 5.98%, and 7.18%, respectively. The invasion rates of CC7 strains with truncated *inlA* into Caco-2 cells were significantly higher than those of *inlA*-carrying strains (Figure 7B). In CC193, the invasion rate of the *inlA*-carrying strain 21212 was 0.46%, which was considerably lower than that of the *inlA* with PMSC strain 21302* (12.86%). No significant difference was observed in the adhesion rates between 21196 and 21203.

## 4. Discussion

Molecular typing methods are crucial for tracking outbreaks to prevent and control disease transmission during surveillance of listeriosis. In this study, whole-genome sequencing (WGS) was used to analyze the *inlA* gene of 546 *L. monocytogenes* strains, and their potential virulence was investigated based on the diversity of *inlA*. The pathogenicity of *L. monocytogenes* varies among different clonal complexes (CCs). Previous epidemiological studies have shown that CC6, CC1, and CC2 are closely associated with clinical cases in the United States and European countries, while CC87, CC8, CC5, and CC3 are the most common sequence types in human infections in China [24,25,26]. In our study, CC155, CC87, CC5, CC3, and CC2 all carried intact *inlA*, while only one strain in CC8 (1/90) harbored a truncated *inlA*. Additionally, we found that among all 27 CCs, isolates from six CCs had premature stop codon (PMSC) mutations (Figure 1). Notably, CC9 and CC121 strains had the highest number of *inlA* with PMSCs, consistent with previous studies [17,27]. However, CC9 and CC121 strains are rarely isolated from clinical cases [24] and are considered low-virulence clones found in food and related environments, particularly in meat processing plants [28]. Although truncated InlA is often associated with attenuated virulence [29], PMSCs are still detected in approximately 3% of clinical strains carrying InlA [17]. Therefore, infection with *L. monocytogenes* strains carrying truncated InlA can be life-threatening for immunocompromised individuals.

Previous studies have shown that the full-length *inlA* profile is more prevalent in cold-, salt-, and acid-resistant strains than in strains that are sensitive to these conditions [30]. Therefore, full-length *inlA* may be involved in the stress response of *L. monocytogenes*. To evaluate the effect of *inlA* on the in vitro biological characteristics of *L. monocytogenes*, *inlA* was knocked out in the wild-type strain LM119 by homologous recombination to obtain the deletion strain LM119-Δ*inlA*. Subsequently, the growth capacities of both strains were tested. The results showed that *inlA* deletion did not affect the growth capacity of LM119, suggesting that this gene may not be essential for the normal growth of *L. monocytogenes*. Because the growth capacity of the mutant strain remained unchanged, subsequent studies on the resistance and virulence of the mutant strain do not have to consider the influence of differences in the strain’s growth capacity.

Biofilms allow *L. monocytogenes* to withstand various environmental stressors [31]. In previous studies, PrfA, which positively regulates numerous virulence genes, was reported to promote biofilm formation, as strains overexpressing PrfA exhibited a higher biofilm-forming ability than wild-type strains [32]. Studies have shown that the presence of truncated InlA protein is significantly associated with increased biofilm formation [33,34]. When bacteria are exposed to adverse conditions, their cell envelope is the first line of defense. Therefore, the absence of the cell wall-anchored InlA protein may alter cell surface characteristics, making cells more vulnerable to specific environmental stresses. However, we did not observe any significant difference in the biofilm-forming ability between LM119 and LM119-Δ*inlA* at either room temperature or 37 °C (closer to human body temperature), indicating that *inlA* deletion does not affect biofilm formation by *L. monocytogenes* at these temperatures.

During *L. monocytogenes* infection, pathogens must withstand the gastrointestinal environment—including low pH and bile salts—as they enter the digestive system with food to infect intestinal cells [22]. Therefore, we also investigated the effect of *inlA* gene deletion on the tolerance of *L. monocytogenes* to digestive fluids (Figure 5). Overall, SGF and SIF exhibited bactericidal activity, with SGF exhibiting bactericidal activity, consistent with previous findings [35]. After 1 h of treatment in SGF, the bacterial concentrations of LM119 and LM119-Δ*inlA* decreased by 1.76 ± 0.21 log CFU/mL and 1.73 ± 0.14 log CFU/mL, respectively, with no significant difference in bacterial reduction between the two strains (*p* ≥ 0.05). Our results indicate that *inlA* deletion does not affect the strain’s tolerance to gastrointestinal fluid. Previously, Hadjilouka et al. [36] performed reverse transcription quantitative PCR on *L. monocytogenes* exposed to gastric and intestinal fluids to assess the transcription of virulence genes. They observed no upregulation or downregulation of the virulence gene, *inlA*. Overall, our results are mutually consistent with the findings of this study.

The *inlA* gene in the reference strain EDG-e of *L. monocytogenes* encodes a full-length InlA protein consisting of 800 amino acids [11]. This protein is divided into the following regions: 1aa–34aa is the signal sequence (SS), 35aa–77aa comprises the α-helix region, 78aa–413aa is the LRR, 414aa–517aa is the immunoglobulin-like intergenic repeat region (IR), 518aa–707aa is the B-repeat region, 707aa–766aa is the marker sequence essential for Gram-positive bacterial sorting and cell wall anchoring (Protein A, PA), and 767aa–800aa is the Leu-Pro-X-Thr-Gly C-terminal cell wall anchor motif (LPXTG) [11]. LRR and LPXTG are two important domains of InlA. LRR is highly conserved and facilitates the interaction between InlA and the human surface receptor E-cadherin and is reported to be highly conserved [37]. In LPXTG, X can be any amino acid, and this domain is covalently anchored to the bacterial surface through peptidoglycan.

Analysis of the *inlA* gene suggests that the presence of PMSCs may interfere with the invasiveness of strains, depending on their nucleotide positions. Studies have reported that the presence of a stop codon at nucleotide position 976 (*inlA*-69) does not hinder the adhesiveness and invasiveness of the isolates [16]. Unfortunately, this study did not provide information on *L. monocytogenes*, such as CC types. In our research, the *inlA*_69 type was only found in two CC types, CC9 and CC121. CC9 and CC121 are known to be low-virulence *L. monocytogenes* strains, as evidenced by their low adhesion and invasiveness to Caco-2 cells. There was no significant difference between *inlA* with PMSC and intact *inlA* strains. Similar results have been reported: Jacquet et al. [38] showed a C-T transition at position 565 (*inlA*_40, *inlA*_1133, 1134), Van Stelten & Nightingale [39] and Van Stelten et al. [18] showed G–T at position 229 (*inlA*_35), G–T at position 508 (*inlA*_1143), and T–A at position 758 (*inlA*_1144) did not hinder the invasion and adhesion of *L. monocytogenes* into Caco-2 cells.

However, this study showed that in two highly virulent CC types, the adhesion of *inlA* to PMSC strains from CC8 to Caco-2 cells was significantly increased (*p* < 0.05), but the invasiveness of CC8 and CC7 was significantly decreased (*p* < 0.05). The *inlA* allele types in CC8 and CC7 were *inlA*_48 and *inlA*_49, respectively. However, the same PMSC types in low-virulence CC9 and CC121 strains showed no significant differences in adhesion or invasiveness to Caco-2 cells. Based on these results, a preliminary conclusion is that the effect of *inlA* with PMSC on the adhesion and invasiveness to Caco-2 cells is less related to PMSC itself and more related to the CC type of *L. monocytogenes*, which is, in turn, strongly associated with its virulence. In this study, CC9 and CC121 were low-virulence strains, while CC8 and CC7 were moderately and highly virulent strains, respectively [40].

The conclusions of this study complement those of previous literature, indicating that *inlA* plays an important role in the virulence of *L. monocytogenes*, although it is not the sole determinant [41,42]. Other factors associated with invasion, including listeriolysin O (LLO), may act synergistically to enhance host invasion efficiency [10]. *InlA* and LLO are the most critical invasion factors, although their roles differ depending on the cell type [10]. In other words, the change in virulence of *L. monocytogenes* caused by *inlA* mutations is one of the factors, and the roles of other virulence genes and regulatory genes cannot be ignored. For instance, in our invasion and adhesion experiments, the results of virulence significance analysis between two *inlA*-carrying strains in CC193 and the strain carrying *inlA* with PSMC (21196 vs. 21302* and 21212 vs. 21302*) were not entirely consistent. A similar phenomenon was observed by Ferreira et al. [29]. They found that a clinical isolate (1547) exhibited impaired ability to invade Caco-2 cells, yet it had a full-length InlA, and the transcript level of *inlA* was significantly higher than that of the control strain. These results all indicate that the strain is likely to carry other virulence-related genes, which lead to the impairment of epithelial cell invasion ability.

During the adhesion of *L. monocytogenes*, cellular invasion is promoted by the activation of signaling cascades in host cells via bacterial surface adhesion factors [43]. Each adhesion phase depends on several bacterial factors acting synchronously, which are in turn regulated by specific regulatory genes [43]. These orchestrated interactions involve the participation of virulence factors that are specifically regulated at transcriptional, post-transcriptional, and post-translational levels [43]. The main virulence factors involved in adhesion are Lap, Ami, DltA, FbpA, InlJ, CtaP, LapB, ActA, RecA, and InlF. Factors involved in invasion include InlA, InlB, Vip, Auto, P60, Lgt, GTCA, LpeA, MprF, LLO, Flagella, and ActA [43]. Thus, multiple virulence factors may be involved in the same stage of the infection cycle, with some acting at the same phase and others having more limited roles.

Therefore, adhesion and invasiveness are complex processes involving *inlA* and other virulence-related factors, such as *inlB*. Our study preliminarily showed that premature termination of *inlA* did not significantly affect the adhesiveness and invasiveness of low-virulence CC-type *L. monocytogenes* strains to Caco-2 cells, but significantly influenced the adhesiveness and invasiveness of higher-virulence strains such as CC8 and CC7. Therefore, it can be further concluded that the impact of *inlA* with PMSC types on virulence is related to the virulence of *L. monocytogenes* itself, which is strongly associated with CC types. Thus, expanding the number of strains with different CCs may aid in unraveling the association between PMSCs and CC types and help determine whether a PMSC affects the invasion of Caco-2 cells. Furthermore, other potential mechanisms of bacterial adhesion and invasion should be investigated. Nevertheless, based on the cell adhesion and invasion abilities of the *L. monocytogenes* isolates shown in this study, we can conclude that these strains may pose a potential public health risk.

## 5. Conclusions

As an important virulence factor of *L. monocytogenes*, a comprehensive understanding of the polymorphism, truncation types, and distribution characteristics of the internalin *inlA* gene will significantly benefit the study of the pathogenicity of this bacterium. This study found that diverse forms of truncated *inlA* exist in *L. monocytogenes* strains. Moreover, *inlA* with PMSCs was associated with CCs, of which CC9 and CC121 were predominant. In addition, our research revealed that while *inlA* did not affect the resistance of *L. monocytogenes*, it played a critical role in the adhesion and invasiveness of the bacteria into Caco-2 cells. The virulence of *L. monocytogenes* strains carrying *inlA* or *inlA* with PMSC was associated with their CC type. Our study preliminarily showed that premature termination of *inlA* had no significant effect on the adhesion to and invasion of low-virulence CC strains into Caco-2 cells but significantly influenced that of higher-virulence strains, such as CC8 and CC7. In summary, this preliminary study highlights the impact of *inlA* integrity and PMSC variations across different CCs on the virulence of *L. monocytogenes*, paving the way for further research on *inlA*-related pathogenic mechanisms.

## Figures and Tables

**Table 1:** Information on *L. monocytogenes* strains used in virulence and resistance experiments.

Number	Strains	CC Types	*inlA* Types
1	119	87	*inlA*
2	119-Δ*inlA*	87	-
3	142	121	*inlA*
4	143	121	*inlA*
5	144	121	*inlA*
6	239*	121	*inlA*-49
7	20109	9	*inlA*
8	2249*	9	*inlA*-49
9	145*	9	*inlA*-43
10	126	8	*inlA*
11	180	8	*inlA*
12	181	8	*inlA*
13	2298*	8	*inlA*-49
14	015	7	*inlA*
15	003	7	*inlA*
16	030	7	*inlA*
17	016*	7	*inlA*-48
18	21212	193	*inlA*
19	21196	193	*inlA*
20	21302*	193	*inlA*-41

* Represents premature termination of *inlA* carried by the strain; - Indicating that the strain does not carry *inlA.*

**Table 2:** Frameshifts and mutations causing premature stop codons (PMSCs) in *inlA* identified in this study.

Allele	Mutant Site	InlA Amino Acid Length (aa)	CCs/Number
48	12 (deletion A)	8	CC9/68, CC121/1, CC7/1
49	1474 (C → T)	491	CC9/2, CC121/61, CC8/1
47	1637 (deletion A)	576	CC9/19, CC121/1, CC321/1
69	976 (G → T)	325	CC9/23, CC121/1
43	1380 (G → A)	459	CC9/5
44	2054 (G→ A)	684	CC9/1
688	262 (C → G)	55	CC121/1
91	2100 (C → G)	699	CC321/8
41	12 (insertion A)	25	CC193/3

## Data Availability

The original contributions presented in the study are included in the article/Supplementary Material, further inquiries can be directed to the corresponding authors.

## Supplementary Materials

The following supporting information can be downloaded at: https://www.mdpi.com/article/10.3390/foods14172955/s1. Table S1. Primer sequences required for this experiment.
