# Supplementary material for: Research on the Genetic Polymorphism and Function of inlA with Premature Stop Codons in Listeria monocytogenes
Source: Foods. 2025 Aug 25;14(17):2955. doi: 10.3390/foods14172955 (PMC12427964; doi:10.3390/foods14172955)
Supplement: Supplementary file 1 [file foods-14-02955-s001.zip › foods-3738258-supplementary.pdf]

Table S1. Primer sequences required for this experiment

Primers	Primer sequence (5'-3')	Purpose
up-inlA.F	CCTGCAAGTGGCACAATATCACTAATTTGG	Amplify the upstream fragment A of *inlA*
up-inlA.R	GCACGTGCTAGTAAATAGGAAGTAGTGTAAAGAGCTAGATGTGG	
down-inlA.F	ccgctgccaaatactaatattgc	Amplify downstream fragment B of *inlA*
down-inlA.R	gccaaccgtggtaaatggaaaaaacct	
2up-inlA.F	CCTGCAAGTGGCACAATATCACTAATTTGGTTATCTTCG	Integrating upstream and downstream sections A and B
2up-inlA.R	CTTCCTATTTACTAGCACGTGCccgctgccaaatactaat	
2down-inlA.F	CTTCCTATTTACTAGCACGTGCccgctgccaaatactaat	
2down-inlA.R	gccaaccgtggtaaatggaaaaaaccttgaactg	
AB-16.F	gggaacaaaagctggtaccCCTGCAAGTGGCACAATATC	Constructing pLR16 pheS * -Δ*inlA*
AB-16.R	ccatttaccacggttggcggatccactagttctaga	
inlA.F	gtgagaaaaaaacgatatgtatggttgaaaagtatactagt	Verify if the target gene inlA is missing
inlA.R	ctatttactagcacgtgcttttttagtaagagc	
